# Short- to mid-term clinical outcomes and return to sport and work after arthroscopic debridement of the extensor carpi radialis brevis in patients with chronic lateral epicondylopathy

**DOI:** 10.1007/s00402-026-06456-4

**Published:** 2026-08-03

**Authors:** Sebastian Siebenlist, Alexandros Doucas, Franziska L. Hatt, Sebastian Lappen, Romed P. Vieider, Lucca Lacheta, Pavel Kadantsev

**Affiliations:** 1 Department of Sport Orthopaedics, Rechts der Isar Hospital, Munich, Germany; 2 Department of Orthopaedic Surgery, Universitätsklinik Balgrist, Zurich, Switzerland; 3 Department of Orthopaedic and Trauma Surgery, University Medical Centre Mannheim, Mannheim, Germany

**Keywords:** Elbow arthroscopy, Lateral epicondylitis, Tennis elbow, ECRB-debridement, Extensor carpi radialis brevis debridement

## Abstract

**Background:**

This study aimed to evaluate the short- to mid-term clinical outcomes following arthroscopic extensor carpi radialis brevis (ECRB) debridement in patients with chronic epicondylopathy humeri radialis (cEHR). We hypothesized that arthroscopic ECRB debridement would result in favorable short- to mid-term patient-reported outcomes, improving pain levels, strength, range of motion (ROM) and patient satisfaction.

**Methods:**

A retrospective case series was conducted on patients who underwent arthroscopic ECRB debridement for cEHR with symptoms > 6 months. Patients were assessed at a minimum of 12 months postoperatively using the Mayo Elbow Performance Score (MEPS), Patient-Rated Tennis Elbow Evaluation (PRTEE), Disabilities of the Arm, Shoulder, and Hand (DASH) score, and Visual Analog Scale (VAS) for pain. Pre- and postoperative scores were compared using DASH and VAS. Maximum isometric wrist extension strength and postoperative ROM were measured bilaterally. Return to sport and work, patient satisfaction, and complications were documented.

**Results:**

Of the 35 eligible patients, 30 completed follow-up (86% follow-up rate; mean age 48.4 ± 6.6 years; range: 36–65 years; 47% male). The mean duration of symptoms before surgery was 12 months (IQR 8–20, range 6–62). The mean follow-up duration was 28.13 ± 10.9 months (range 12–45). At final follow-up, median outcome scores were 100 (IQR 100–100; range 65–100) for the MEPS, 8.7 (IQR 0.8–13.9; range 0-39.2) for the DASH, 0 (IQR 0–1; range 0–7) for the VAS and 10.3 (IQR 0.8–26.8; 0–79.5) for the PRTEE. In comparison to preoperative values, VAS scores significantly improved (6 [IQR 2.75–7, range 9] vs. 0 [IQR 0–1, range 0–7], *p* < 0.01), as did DASH scores (46.6 ± 20 vs. 10.3 ± 10.5, *p* < 0.01). Post-operatively, all patients recovered full range of motion, with an arc of at least 140°. The median wrist extension strength in the operated arm was 98.6% (IQR: 87.4–107%, range: 69.5–121%) when compared to the healthy side, with no significant differences (*p* = 0.681). Overall, 80% of patients returned to sports at the same or a slightly reduced level, including 12% who achieved a higher activity level than before. The vast majority of patients (97%) successfully returned to their previous professional activities, while only a small proportion (3%) retired from a part-time manual labor position post-surgery, primarily due to age. Furthermore, 83% of patients indicated they would undergo the procedure again. One patient developed posterolateral rotational instability six months after index surgery and consequently required reconstruction of the lateral ulnar collateral ligament (LUCL).

**Conclusion:**

Arthroscopic ECRB debridement for cEHR was associated with improved pain and function and high rates of return to sport and work at short- to mid-term follow-up.

**Level of evidence:**

IV Retrospective case series.

## Introduction

 Chronic epicondylopathy humeri radialis (cEHR) is widely regarded as the most common cause of elbow pain [5, 18, 26, 27]. Typically, it is self-limiting, with most patients becoming asymptomatic within one year of symptom onset [5, 18, 26, 27]. Initial treatment is recommended to be nonoperative, focusing on rest, physical therapy, nonsteroidal anti-inflammatory drugs (NSAIDs), bracing, and extracorporeal shock wave therapy. Injection therapies, including autologous blood, corticosteroids, or platelet-rich plasma, may also be considered [9, 10, 28]. Nevertheless, 4–11% of individuals continue to have symptoms despite thorough conservative management [18, 30]. In cases of therapy-resistant cEHR, surgical intervention - regardless of the technique employed - typically yields favorable outcomes, particularly in terms of pain relief and patient satisfaction [12, 13, 18]. Based on current data, it is not yet possible to make an evidence-based recommendation for a specific surgical procedure [13, 16, 18].

The extensor carpi radialis brevis (ECRB) tendon is the primary structure affected in cEHR, primarily due to repetitive stress and overuse [19]. Histopathological analyses have identified a degenerative process in the ECRB tendon, marked by fiber microtears, angiofibroblastic hyperplasia, fibrosis, disorganized loose collagen, and osseous calcifications. These pathological changes may contribute to the progressive fibrocartilaginous transformation of the ECRB enthesis, weakening the tendon-bone junction and compromising its structural integrity [1, 6, 8, 19]. Those changes may contribute to the failure of conservative treatment.

Arthroscopic ECRB debridement has been introduced to be one option for the treatment of cEHR [7, 12]. It allows a minimal invasive approach to treat chronic epicondylopathia and if necessary, management of additional intra-articular pathology. The arthroscopic approach offers significant advantages over open and percutaneous procedures [30]. The ability to perform direct visual assessment of stability and integrity of the lateral capsule complex, lower rates of wound healing complications and the favorable cosmetic outcome with smaller skin incisions are among the major advantages of an arthroscopic approach when compared to open techniques [12, 22].

Therefore, the aim of this study was to evaluate the clinical outcomes and rates of return to sport and work following arthroscopic ECRB debridement in patients with therapy-resistant cEHR of the elbow. We hypothesized that this procedure would result in favorable functional outcomes in terms of a high rate of successful return to work, and a low failure rate over short- to mid-term follow-up. Additionally, we hypothesized that this treatment modality would facilitate a high rate of return to sports.

## Materials and methods

A retrospective analysis was conducted on consecutive patients who underwent arthroscopic ECRB debridement between November 2020 and January 2024 at a single center by a single surgeon (S.S.). Institutional review board approval was obtained prior to this study (2023-279-S-KH) and the study was conducted according to the Declaration of Helsinki. Informed consent was obtained by each individual prior to the clinical evaluation.

### Patient selection

Included were patients who failed conservative management for cEHR (symptom duration > 6 months) and thus were treated by arthroscopic ECRB debridement. Eligibility criteria required patients to be over 18 years old. Patients with concomitant pathologies of the elbow (cartilage defects, free joint bodies, instability), previous surgeries of the affected elbow or relevant comorbidities such as rheumatic diseases, cervical and peripheral neuropathies, malignant tumor diseases, and metabolic diseases were excluded. Additional information such as demographic data including age, sex, and hand dominance as well as sports participation were collected. Intraoperative and postoperative complications were reported.

### Surgical technique - ECRB debridement

**Fig. 1:**
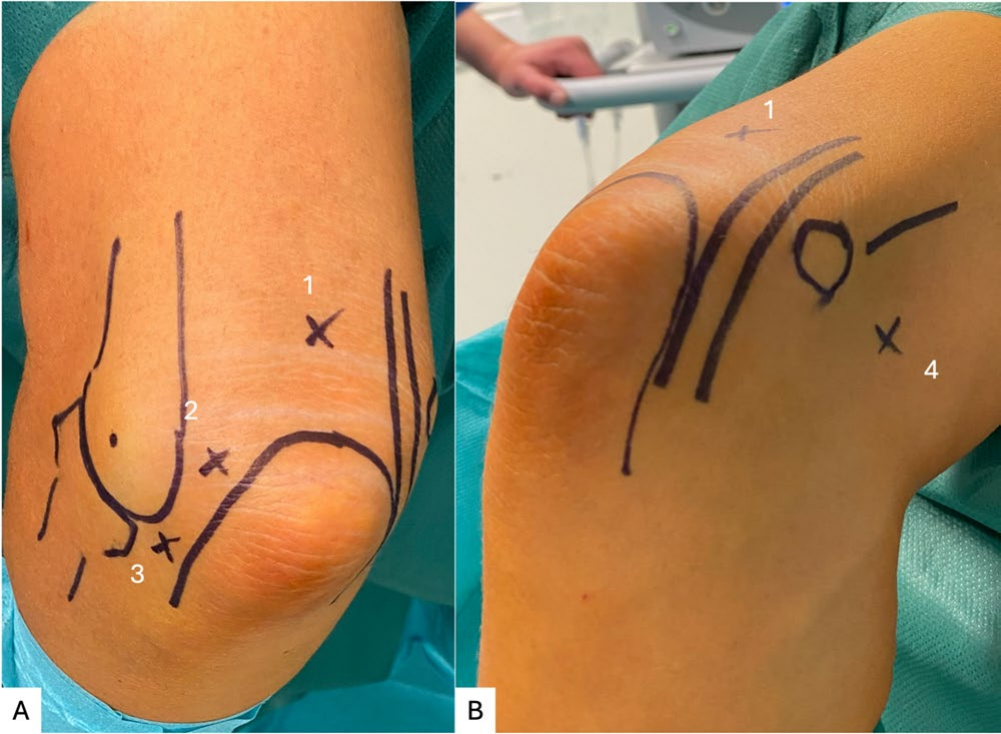
Preoperative marking of the anatomical landmarks. **A** View from the radial side: radial epicondyle, radial head, and olecranon with the postero-central (trans-tricipital) portal [1], the high postero-radial portal [2], and the soft-spot portal [3]. **B** View from the ulnar side: ulnar epicondyle, ulnar nerve, and olecranon with the postero-central [1] and antero-medial portal [4]

**Fig. 2:**
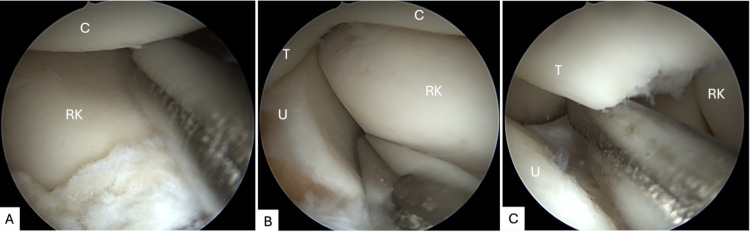
Stability test with a switching rod. **A** Radiocapitellar joint, **B** Proximal radioulnar joint, **C** Humeroulnar joint. Anatomical Structures: C – Capitellum. RK – Radial head. T – Trochlea humeri. U – Ulna

**Fig. 3:**
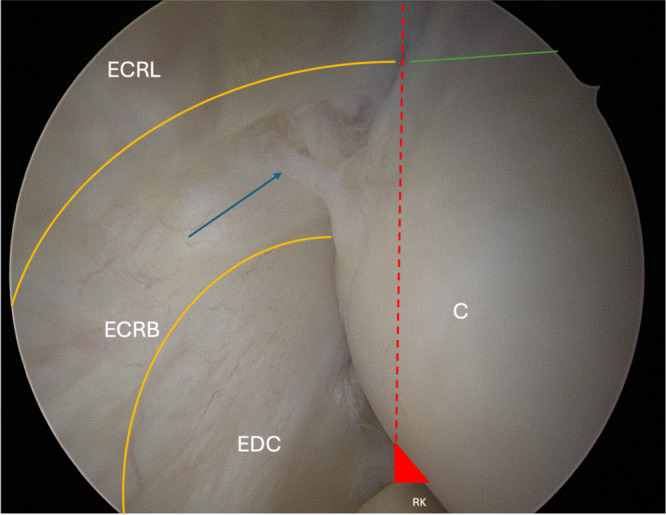
An anteromedial view of the lateral capsular attachment with the underlying tendon insertions of the extensor carpi radialis longus (ECRL), extensor carpi radialis brevis (ECRB), and extensor digitorum (EDC). The red line marks the equator of the radial head, and the red shading indicates the posterolateral joint capsule. Debridement posterior to the radial head should be avoided! C – Capitellum. RK – Radial head. Blue arrow – Capsular defect. Green line – Cartilage-bone transition of the capitellum

**Fig. 4:**
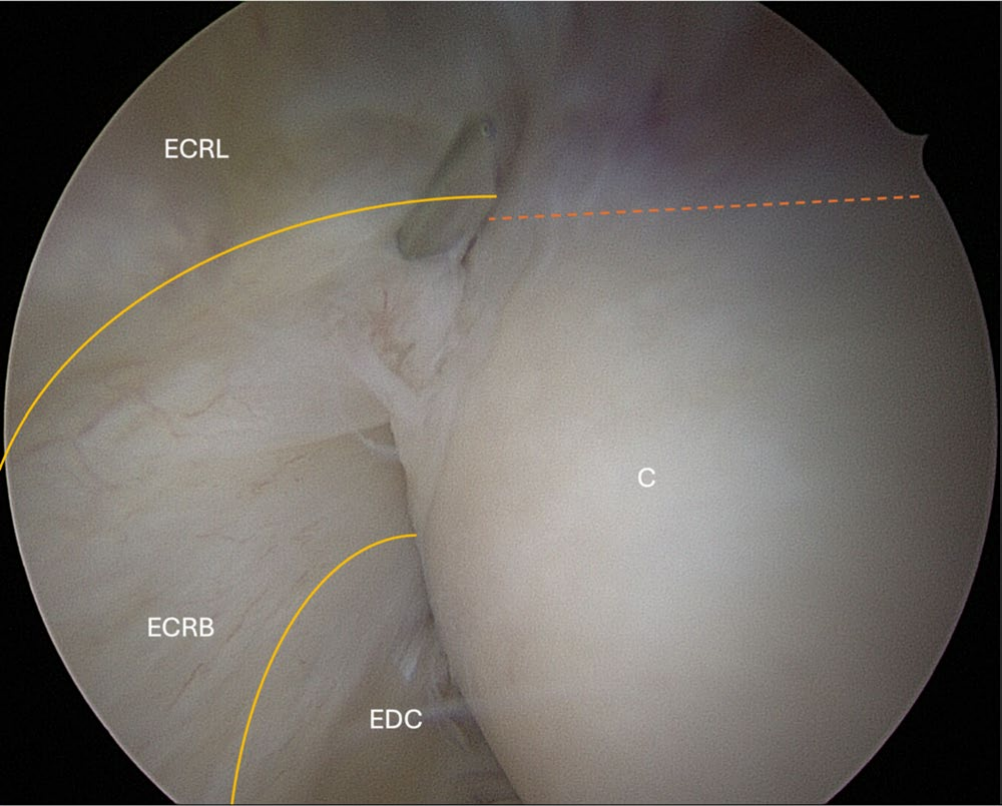
Creation of the anterolateral working portal using the “Needle & Knife” technique under direct visualization at the cartilage-bone junction of the capitellum. C – Capitellum. ECRB – Extensor carpi radialis brevis. Dashed line – Cartilage-bone junction of the capitellum

**Fig. 5:**
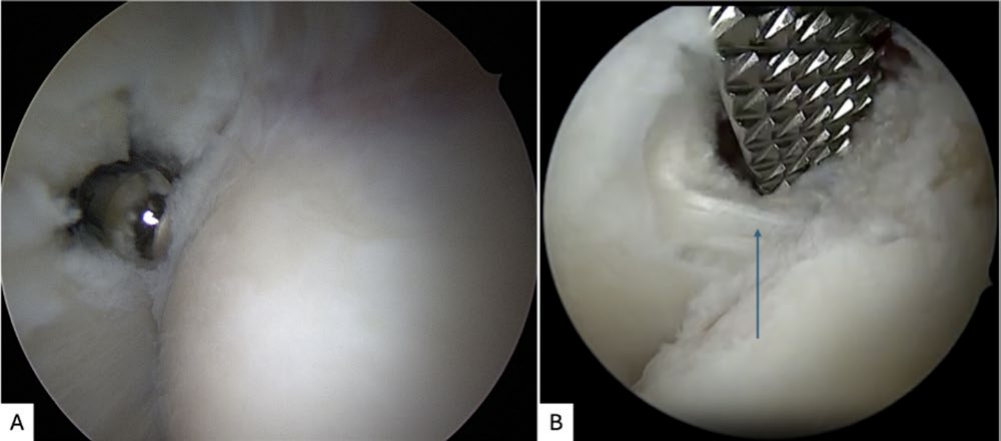
**A** ECRB debridement using a shaver. **B** Afterwards, the bone in the area of the ECRB insertion is freshened using a raspatory (blue arrow)

The patient is routinely positioned in the lateral decubitus position. The arm is positioned and secured at 90° shoulder abduction and 90° elbow flexion, ensuring that a full range of elbow motion is possible throughout the procedure. A tourniquet (220 mmHg) is routinely applied to the proximal humerus. After joint distension with 20-40 ml saline, an inflow cannula is inserted through the anteromedial portal. A standardized diagnostic examination is performed to rule out concomitant pathologies. Humeroradial, humeroulnar and radioulnar stability were assessed by a switch rod to assure joint congruency. The anterior compartment of the joint is inspected through the anteromedial portal. Via the anteromedial portal a direct visualization of the lateral capsule and radiocapitellar joint is ensured, allowing detection of capsular defects or tears. An anterolateral working portal is created under direct visualization at the ECRB insertion site, marked and created using the needle-and-knife technique. The capsule overlying the ECRB is carefully removed with an oscillating shaver to gain access to the extracapsular ECRB attachment. Pathological tissue at the proximal origin of the ECRB is debrided until spontaneous bleeding is observed. Special care is taken to maintain the relationship to the equator of the capitellum to avoid injury to the lateral ulnar collateral ligament (LUCL). (Figures 1, 2, 3, 4 and 5)

From the first post-operative day, full range of motion of the elbow is allowed within a pain-free range, and normal daily activities are allowed. However, physical labor and sports activities should be avoided for the first six weeks postoperatively. Pain and swelling management included pharmacological therapy (NSAIDs), cooling, elevation and, if necessary, manual lymphatic drainage of the elbow joint. Physiotherapy was also recommended to prevent joint stiffness up to 6 weeks postoperatively.

### Patient-reported outcome

Functional outcome was assessed using the Mayo Elbow Performance Score (MEPS), the Patient Rated Elbow Evaluation (PRTEE) at final follow-up. The questionnaire on disabilities of the arm, shoulder and hand (DASH) and the current pain status using a visual analog scale (VAS) with a rating range from 0 (“no pain”) to 10 (“worst pain”) were additionally assessed.

### Functional outcome

Range of motion (ROM) of both elbows using a goniometer was documented. Postoperatively isometric muscle strength tests for the wrist extension (IsoBex; MDS Medical Device Solutions AG, Oberburg, Switzerland) were performed on both arms. Maximum wrist extension strength was measured at 90° elbow flexion with the forearm in neutral rotation and stabilized on the table surface [17]. Three consecutive measurements were obtained for each patient and then averaged. To accurately assess postoperative differences in strength relative to the healthy side, strength ratios were calculated for each patient. For this purpose, the wrist extension strength of the operated side was divided by the respective strength of the non-operated side (limb symmetry index), with final values being presented as percentages.

### Return to sports/work rate and patient satisfaction

Patients’ sporting activities before symptom onset and at follow-up were assessed by a customized questionnaire to evaluate the return-to-sport rate by four grades “higher level”, “similar level”, “slightly reduced level” or “reduced level”. A return-to-work (RTW) questionnaire was administered to assess occupational outcomes. Patients were asked to report their employment status (employed, self-employed, performing housework, retired, or unemployed) and average weekly working hours (0; 0–10; 10–20; 20–30; 30–40; or > 40 h), both preoperatively and at one year postoperatively. Completion of both the return-to-sport (RTS) and RTW questionnaires, in accordance with the provided instructions, was required for inclusion in the final analysis. Additionally, patients were asked to rate their current functional status and satisfaction with the surgical outcome, and to report whether any further surgical interventions had been performed.

### Complications

Peri- and postoperative complications were documented and reported.

### Statistical analysis

All calculations were performed with SPSS Statistics (Version 25, Property IBM Corp., NY, USA). The normality of the data distribution was assessed using the Kolmogorov‒Smirnov test. Paired and unpaired t-tests were used to analyze normally distributed data. The Wilcoxon test and the Mann-Whitney U test were used to analyze data that did not fulfill normal distribution. The level of significance was set at *p* < 0.05. To complement p-values and estimate the clinical magnitude of pre- to postoperative changes, effect sizes were calculated. For parametric comparisons, Cohen’s d was used and interpreted as small, moderate, or large for values of 0.2, 0.5, and 0.8, respectively. For non-parametric comparisons, effect size r was calculated as Z/√N and interpreted as small, moderate, or large for values of 0.1, 0.3, and 0.5, respectively.

## Results

### Demographics

Of the 35 eligible patients who were included in this study, 30 (86%) were available for final follow-up. Five patients were lost to follow-up due to outdated contact information. Four patients were unable to attend the clinical follow-up appointment and were therefore evaluated using questionnaires and telephone interviews. The mean follow-up was 28.13 ± 10.9 months (range 12–45). The cohort consisted of 14 male and 16 female patients with an average age of 48.4 ± 6.6 (min: 36; max: 65) years. The median interval between symptom onset and surgical treatment was 12.0 months (IQR 8–20 range: 6–62). Twenty-two patients (73%) underwent preoperative evaluation using the DASH and VAS scales. 25 patients reported participating in high-demanding sports such as weightlifting, tennis, climbing or throwing sports.

### Patient-reported outcome

The following outcome measures were observed at final evaluation: MEPS 100 (IQR 100–100, range 65–100); DASH 8.7 (IQR 0.8–13.9, range 0–39.2); VAS 0 (IQR 0–1, range 0–7); PRTEE 10.3 (IQR 0.8–26.8, range 0–79.5).

**Fig. 6:**
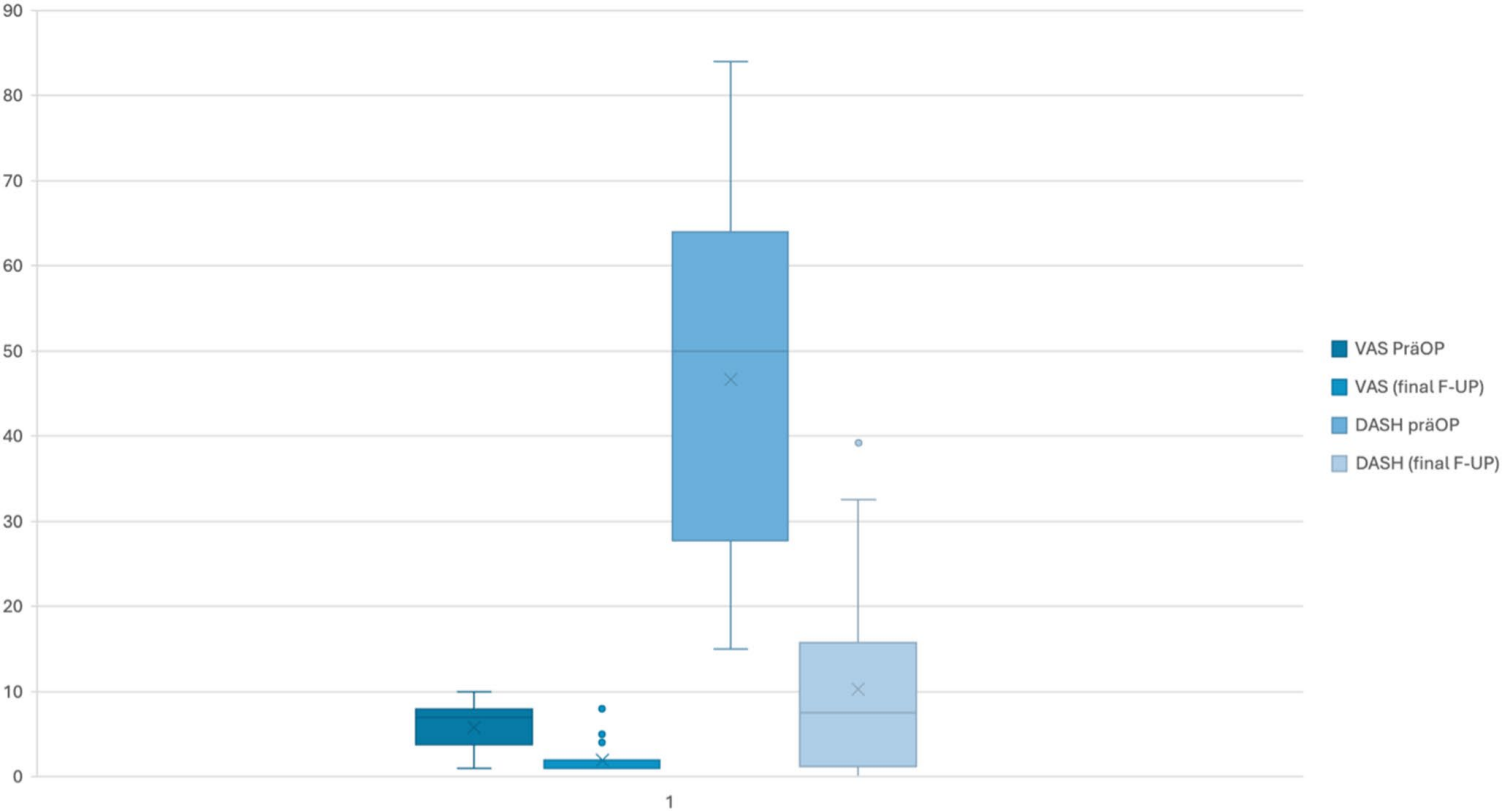
Comparison of pre- and post-operative assessment of VAS and DASH in 22 patients

Pre- to postoperative assessment using the DASH and VAS scales was conducted for 73% of patients. (Fig. 6) Table 1 shows the significant improvement of both values after surgery. In addition to statistical significance, the pre- to postoperative improvements were clinically meaningful, with a very large effect size for the DASH score (Cohen’s d = 2.02) and a large effect size for VAS pain reduction (*r* = 0.65).

A significant improvement in pain was reported by 91% of patients following surgery. A small proportion (6%) experienced no change in pain levels. The only patient (3%) who experienced worsening pain had pre-existing, therapy-resistant medial epicondylitis in addition to cEHR before surgery. Moreover, their demanding work schedule prevented adherence to postoperative recommendations for proper loading of the operated arm.

**Table 1:** Comparison of pre- and post-operative assessment of 22 patients

Parameter	Preoperative	Postoperative	*p* value
DASH	46.6 ± 20	10.3 ± 10.5	< 0.01*
VAS	6 (IQR 2.75–7, range 0–9)	0 (IQR 0–1, range 0–7)	< 0.01*

DASH disabilities of the arm, shoulder and hand, VAS visual analog scale

* denotes statistical significance with a p-value < 0.05

### Functional outcome

Post-operatively, all patients recovered full range of motion, with an arc of at least 140°. The median strength of the operated arm, relative to the healthy side, was 98.6% (IQR: 87.4–107%, range: 69.5–121%) for wrist extension. The strength differences between the healthy and affected arms were negligible and not statistically significant (*p* = 0.681).

### Return to work and sports rate and patient satisfaction

Most patients (97%) successfully returned to their previous professional activities, while a very small proportion (3%) retired from a part-time manual labor position post-surgery, primarily due to age. Overall, 79% of patients resumed work within six to twelve weeks, with more than half of them (56%) returning within six weeks. Notably, all patients returned to full-time employment.

All patients who regularly participated in sports before symptom onset successfully returned to their activities following surgery. Among them, 68% regained a similar or slightly reduced level of sporting activity, while 12% achieved an even higher level than before the operation. Regarding the time required to return to sporting activity, 16% of patients resumed sports within 6 weeks after surgery, 36% within 3 months, 36% within 6 months, and the remaining 12% returned 12 months post-operation.

Regarding subjective satisfaction, 73% of patients were satisfied with the treatment outcome, and 83% stated they would undergo the procedure again. Overall, 27% reported some degree of dissatisfaction. Among these individuals, 50% would still choose surgery again, and half reported no pain at final follow-up.

### Descriptive analysis of dissatisfied patients and non-responders

A descriptive subgroup analysis was performed to compare satisfied and dissatisfied patients. Dissatisfied patients did not show an obvious systematic difference from satisfied patients regarding age, sex distribution, BMI, preoperative DASH or VAS scores, or preoperative sports participation. However, dissatisfied patients showed less favorable patient-reported postoperative outcomes, with higher PRTEE scores, higher postoperative DASH scores, and higher VAS pain scores. In a descriptive subgroup analysis, dissatisfied patients showed a higher mean symptom duration before surgery than satisfied patients, although the median symptom duration was 12 months in both groups. Mean symptom duration was 22.0 ± 20.6 months in dissatisfied patients compared with 15.6 ± 11.7 months in satisfied patients. Regarding postoperative sport level, dissatisfied patients tended to report lower return-to-sport levels than satisfied patients. Among patients who had participated in sport preoperatively, 3 of 7 dissatisfied patients (42.9%) returned to the same or a higher level, compared with 11 of 19 satisfied patients (57.9%). Because of the small number of dissatisfied patients, these findings were interpreted descriptively and not as definitive predictors of outcome (Tables 2 and 3).

**Table 2:** Descriptive comparison: satisfied vs. dissatisfied patients

	Satisfied	Dissatisfied
Number of patients	22	8
Sex	10 ♂/12 ♀	3 ♂/5 ♀
Age	48.0 ± 8.3 years	49.9 ± 3.3 years
Follow up	29.2 ± 11.7	25.1 ± 8.4
BMI	26.3 ± 4.2	23.9 ± 4.0
Symptom duration	15.6 ± 11.7 months	22.0 ± 20.6 months
Manual/physically demanding jobs	13/22	4/8
Work hours	36.5 ± 14.7 h/week	40.4 ± 6.4 h/week
Sport participation preOP	18/22	7/8
PreOP DASH	48.0 ± 20.3	43.7 ± 20.9
PreOP VAS	6.1 ± 2.4	5.3 ± 2.8
PostOP VAS	0.41 ± 1.05	1.64 ± 2.56
PostOP DASH	7.1 ± 8.1	16.0 ± 11.5
PRTEE	10.7 ± 13.2	31 ± 22.7

Values are presented as mean ± SD or n. Manual/physically demanding jobs were defined as occupations involving repetitive or forceful use of the affected upper extremity, including lifting, gripping, tool use, or sustained wrist extension

**Table 3:** Descriptive comparison: would undergo surgery again vs. would not undergo again

	Would undergo again	Would not undergo again
Number of patients	25	5
Sex	11 ♂/13 ♀	2 ♂/4 ♀
Age	48.4 ± 7.8 years	48.8 ± 5.0 years
Follow up	29.1 ± 11.7	24.3 ± 5.9
BMI	26.2 ± 4.4	23.5 ± 2.9
Symptom duration	16.6 ± 13.0 months	20.2 ± 20.5 months
Manual/physically demanding jobs	16/24	2/6
Work hours	37.8 ± 13.8	36.3 ± 9.8
Sport participation preOP	20/25	5/5
PreOP DASH	49.2 ± 19.1	39.7 ± 22.7
PreOP VAS	6.0 ± 2.4	5.3 ± 2.9
PostOP VAS	0.50 ± 1.38	1.67 ± 2.66
PostOP DASH	9.3 ± 10.4	9.7 ± 5.9
PRTEE	14.4 ± 15.3	23.3 ± 28.1

Values are presented as mean ± SD or n. Manual/physically demanding jobs were defined as occupations involving repetitive or forceful use of the affected upper extremity, including lifting, gripping, tool use, or sustained wrist extension

Patients who stated that they would not undergo the procedure again did not show clear differences in age, sex, BMI, preoperative DASH, or preoperative VAS compared with those who would choose surgery again. However, they showed a tendency toward longer mean symptom duration, higher postoperative pain scores, and higher PRTEE values. Regarding postoperative sport level, patients who stated that they would not undergo the procedure again tended to report lower return-to-sport levels than satisfied patients. Among patients who had participated in sport preoperatively, 1 of 5 patients who stated that they would not undergo the procedure again (20%) returned to the same or a higher level, compared with 13 of 20 patients who stated that they would undergo the procedure again (65%). However, due to the small subgroup size, no reliable statistical conclusions can be drawn.

### Complications

One patient developed persistent lateral elbow pain and progressive instability symptoms after surgery. Preoperative history, clinical examination, and imaging had not shown signs of elbow instability. During the index arthroscopy, no radiocapitellar, humeroulnar, or proximal radioulnar instability was detected, and no relevant intra-articular pathology was observed apart from a capsular dehiscence in the area of the ECRB attachment. According to the operative documentation, debridement was performed according to the described technique, with specific attention to avoiding debridement posterior to the radial equator of the radiocapitellar joint.

At the first documented postoperative follow-up five weeks after surgery, the patient reported being pain-free for the first three postoperative weeks. Approximately four weeks after surgery, the patient reported an overload episode during activities of daily living, followed by new-onset lateral elbow pain. Initially, conservative treatment including physiotherapy, load modification, and NSAIDs was recommended and led to temporary symptom improvement. Three months postoperatively, the patient reported recurrent symptom aggravation. MRI at that time showed postoperative changes around the extensor origin but no clear structural pathology. Because the clinical findings were interpreted as recurrent lateral epicondylopathy, conservative treatment was continued.

At six months postoperatively, persistent lateral elbow pain and progressive clinical symptoms suspicious for posterolateral rotatory instability prompted repeat MRI, which demonstrated partial avulsion of the extensor origin and progressive edema in this region. Revision surgery was discussed with the patient. Because PLRI was suspected clinically, dynamic arthroscopic stability assessment was planned, with LUCL reconstruction if instability was confirmed. During revision surgery, posterolateral instability was confirmed dynamically, and lateral ulnar collateral ligament reconstruction using an ipsilateral triceps tendon graft was performed. No infections or other complications were observed.

## Discussion

The main finding of this study is that arthroscopic debridement of the extensor carpi radialis brevis (ECRB) tendon for the treatment of chronic lateral epicondylopathy (cEHR) yields good functional short- to mid-term clinical outcomes. Patients experienced significant reductions in pain and improvements in DASH, with a high rate of return to both work and sports activities. Additionally, postoperative wrist extension strength closely matched the contralateral side, range of motion (ROM) was preserved, and patient satisfaction was acceptable. The procedure was associated with a low complication and reoperation rate. The MEPS demonstrated a pronounced ceiling effect in the present cohort, with most patients achieving the maximum score at final follow-up.

The descriptive subgroup analysis of dissatisfied patients and patients who would not undergo surgery again suggests that unfavorable subjective outcomes were not clearly associated with age, sex, BMI, preoperative DASH or VAS scores, or preoperative sports/work participation. Instead, dissatisfaction appeared to be more closely related to residual postoperative symptoms, reflected by higher postoperative PRTEE, DASH, and VAS scores. Dissatisfied patients also showed a higher mean symptom duration before surgery. Furthermore, lower postoperative sport level appeared more frequent among dissatisfied patients and particularly among patients who would not undergo surgery again. These findings suggest that residual pain, patient-perceived functional limitation, symptom chronicity, and inability to regain the previous activity level may contribute to dissatisfaction after arthroscopic ECRB debridement. However, because of the small subgroup sizes, these observations should be interpreted as exploratory and not as definitive predictors of outcome.

Compared to conventional open and percutaneous techniques, arthroscopic debridement demonstrates similarly favorable medium- and long-term outcomes in terms of pain reduction, function, complication rates, and reoperation rates [2, 3, 5, 11, 14, 15, 18, 20–22, 29, 31]. Our study findings align with the published literature, confirming that arthroscopic ECRB debridement leads to significant pain reduction and functional improvement in the short- to mid-term follow up [14, 15, 18, 31]. One of the main advantages of arthroscopic ECRB debridement over other surgical techniques is its ability to evaluate and treat concomitant intra-articular pathologies while minimizing disruption of the superficial extensors and ligamentous structures (12, 16).

Peart et al. were the first to report that patients who underwent arthroscopic debridement tended to return to work earlier than those who had open debridement (54 vs. 33 patients, respectively) [21]. These findings have since been reinforced by additional studies [4, 15, 18, 22, 25, 31]. The average return-to-work (RTW) time following arthroscopic ECRB debridement typically ranges between 3 and 8 weeks post-surgery, comparable with the current results [4, 15, 18, 22, 25, 31]. Our study sheds light on the timeline for returning to sporting activities after surgery. All patients who participated in sports prior to the surgery were able to resume their activities, with the majority reaching their pre-symptom level or even surpassing it. This additionally underscores the effectiveness of arthroscopic debridement in a high-demand patient population, demonstrating its suitability as a treatment modality for active individuals.

The case of postoperative PLRI requiring LUCL reconstruction represents the most relevant safety-related event in this series and deserves critical consideration. The reported complication rate for arthroscopic ECRB debridement procedures ranges from 1% to 2.9% [4, 15, 18, 22, 24, 25, 31], . The most common complications include persistent symptoms and iatrogenic injury to the lateral ulnar collateral ligament (LUCL) complex due to excessive debridement [12, 13]. It is therefore essential to have a detailed understanding of the anatomy of the lateral joint region. In the present case, neither the preoperative assessment nor intraoperative stability testing during the index procedure demonstrated elbow instability. The only intraoperative finding in this region was capsular dehiscence at the ECRB attachment. Furthermore, the operative report documented that debridement was performed with attention to avoiding the area posterior to the radial equator of the radiocapitellar joint.

In retrospect, the postoperative course may have suggested evolving lateral-sided pathology, although no definitive clinical signs of PLRI were present during the initial postoperative assessments. After an initially pain-free interval of three weeks, the patient developed new lateral elbow pain following an overload episode approximately four weeks after surgery. Clinical examination at that time showed findings consistent with recurrent lateral epicondylopathy, while specific instability tests did not demonstrate clear posterolateral rotatory instability. Although conservative treatment led to temporary improvement, symptoms later progressed. MRI at three months showed only postoperative changes without a clear structural lesion. In contrast, repeat MRI at six months demonstrated partial avulsion of the extensor origin and progressive edema in this region. The diagnosis of PLRI was ultimately established based on the combination of progressive symptoms, interval MRI findings, and dynamic arthroscopic confirmation during revision surgery.

The observed reoperation rate of 3% in the present cohort is slightly higher than the complication range reported in previous studies. However, this difference should be interpreted cautiously because the cohort size was small and a single event has a substantial effect on percentage-based complication rates. Nevertheless, the clinical relevance of this complication should not be underestimated. This case underlines the need for meticulous anatomical orientation, restrained debridement, and careful intraoperative and postoperative assessment for potential lateral instability. This case led to a further reinforcement of the technical principles used during arthroscopic ECRB debridement. Debridement should remain strictly limited to the pathological ECRB origin, and posterior extension beyond the safe anterior zone should be avoided. Arthroscopy allows for intraoperative assessment of joint stability and direct visualization of the lateral capsule complex, which may be crucial in identifying underlying causes of lateral elbow pain [23]. Intraoperative stability assessment should be performed carefully before and after debridement, especially in patients with capsular defects or dehiscence in the region of the ECRB attachment. A key prerequisite for the success of this minimally invasive procedure is the absence of high-grade structural damage, such as partial avulsion or complete rupture of the extensor tendons. If intraoperative findings reveal extensor avulsion or posterolateral rotatory instability as the primary cause of lateral elbow pain, a seamless conversion to an open procedure (e.g., extensor refixation or ligament stabilization/ reconstruction) can be performed. At the authors’ institution, this possibility is always discussed with patients preoperatively to ensure informed decision-making.

Another secondary benefit of arthroscopic ECRB debridement is better cosmetic outcomes, as it requires smaller incisions compared to open techniques [12, 16, 22]. The incidence of wound infection is significantly lower with arthroscopic procedures (0.74% for open surgery vs. 0% for arthroscopic ECRB-Debridement) [22]. This is also confirmed by the present data.

Proper patient selection and surgical technique are essential for achieving optimal results [12, 13, 18]. While most patients respond well to nonoperative treatment, a small percentage may require surgical intervention. These findings have practical implications for patient selection and preoperative counselling. Patients with concomitant medial epicondylitis or other periarticular pain generators should be evaluated carefully, as residual symptoms may not be fully addressed by isolated ECRB debridement. Similarly, high occupational or athletic loading, prolonged symptom duration, and limited ability to comply with postoperative load restrictions may contribute to less favorable subjective outcomes. Patients should therefore be counselled that arthroscopic ECRB debridement may improve pain and function in many cases, but does not guarantee complete symptom resolution, return to the previous activity level, or full satisfaction. Careful assessment of concomitant pathology, loading demands, symptom chronicity, rehabilitation adherence, and realistic postoperative expectations may help improve patient selection and shared decision-making.

Importantly, cEHR is often self-limiting. Because the present study did not include a nonoperative control group, the observed improvements in pain and function cannot be attributed to arthroscopic ECRB debridement alone. Natural history, rehabilitation, activity modification, placebo effects, and regression to the mean may also have contributed to the favorable outcomes.

This study has several limitations. First, its retrospective design resulted in incomplete preoperative assessment data for eight patients. All procedures were performed by a single experienced elbow surgeon (SS), which may limit generalizability but ensured consistency and standardization of the surgical technique on the other hand. The customized RTS and RTW questionnaires were not formally validated, limiting comparability with other studies. The MEPS showed a clear ceiling effect in this cohort and may therefore have limited sensitivity for detecting residual symptoms or subtle functional limitations. Telephone or questionnaire-based follow-up in four patients may have introduced assessment bias, as objective ROM and strength measurements were not available for these individuals. Preoperative DASH and VAS scores were available for only 22 of 30 patients due to the retrospective design and incomplete standardization of preoperative documentation; therefore, the main pre- to postoperative comparison represents a subgroup analysis, and systematic differences between patients with and without baseline documentation cannot be excluded. Furthermore, the absence of a control group limits comparability with alternative treatment modalities.

Strengths of the study include the reporting of return-to-sport rates in an active patient population and the comprehensive assessment of multiple outcome measures. The availability of preoperative data in a subset of patients allowed retrospective comparison, enhancing the clinical relevance of the findings.

Given that findings of this study align with existing literature and provide new insights into return-to-sport timelines, this study represents a valuable contribution to the ongoing effort to define and standardize the optimal treatment approach for therapy-resistant chronic lateral epicondylitis. To draw more definitive conclusions, high-quality, long-term randomized controlled trials are needed to further establish the most effective treatment modality for recalcitrant chronic lateral epicondylopathy.

## Conclusion

Arthroscopic ECRB debridement for cEHR was associated with improved pain and function and high rates of return to sport and work at short- to mid-term follow-up.

## Data Availability

The datasets generated and/or analyzed during the current study are not publicly available due to institutional and ethical restrictions but are available from the corresponding author on reasonable request and with approval of the responsible institutional review board.
